# Human Mesenchymal Stromal Cells Derived from Perinatal Tissues: Sources, Characteristics and Isolation Methods

**DOI:** 10.21315/mjms2023.30.2.5

**Published:** 2023-04-18

**Authors:** Peik Lin Teoh, Haselamirrah Mohd Akhir, Warda Abdul Ajak, Vun Vun Hiew

**Affiliations:** Biotechnology Research Institute, Universiti Malaysia Sabah, Sabah, Malaysia

**Keywords:** perinatal tissues, mesenchymal stromal cells, surface markers, explant culture, enzymatic method

## Abstract

Mesenchymal stromal/stem cells (MSCs) derived from perinatal tissues have become indispensable sources for clinical applications due to their superior properties, ease of accessibility and minimal ethical concerns. MSCs isolated from different placenta (PL) and umbilical cord (UC) compartments exhibit great potential for stem cell-based therapies. However, their biological activities could vary due to tissue origins and differences in differentiation potentials. This review provides an overview of MSCs derived from various compartments of perinatal tissues, their characteristics and current isolation methods. Factors affecting the yield and purity of MSCs are also discussed as they are important to ensure consistent and unlimited supply for regenerative medicine and tissue engineering.

## Introduction

Stem cells hold a great promise for regenerative medicine and tissue engineering due to their capabilities of self-renewal and differentiation into diverse cell types. They can be generally classified into three types: i) embryonic, ii) adult and iii) foetal. Embryonic stem cells (ESCs) are pluripotent cells derived from the inner cell mass of an embryo. However, controversial ethical issues and teratoma formation after transplantation have hampered the use of ESCs in clinical applications. Later, multipotent stem cells or mesenchymal stromal/stem cells (MSCs) with less ethical constrain from adult tissues such as bone marrow (BM), adipose and dental pulp have become alternative sources despite their limited lineage differentiation potentials compared to ESCs (1). Nonetheless, the invasive procurement of adult stem cells (ASCs) is still a major concern and poses a threat to patients (1, 2).

As a result, MSCs derived from foetal tissues are of growing interests. Both placenta (PL) and umbilical cord (UC) are considered medical wastes, and they do not encompass ethical issues as these tissues are discarded at birth, and the procedures to obtain these tissues are less invasive (3). Foetal tissues are immature cells that have less mutation; thus, the risk of initiating tumorigenesis is reduced (4). They also display better proliferative activity and lower immunogenicity, making them an ideal candidate for cell therapy. Unlike BM-MSCs, the regenerative capability of perinatal MSCs is not dependent on the donor age (5).

According to the guidelines of the International Society of Cellular Therapy (ISCT), the minimal criteria for defining MSCs: i) adhere to laboratory plastic culture; ii) able to differentiate to adipocytes, osteocytes and chondrocytes and iii) express surface markers of CD73, CD105 and CD90 but do not express CD45, CD34, CD14/CD11b, CD79*α*/CD19 and human leukocyte antigen-DR (HLA-DR). MSCs are also found to express CD44, CD106 and HLA class I but not HLA class II. At present, no specific surface marker can be used to isolate MSCs like CD34 for haematopoietic stem cells (6).

Tremendous studies have demonstrated that MSCs from different origins show similarities in the aspects of morphology and immunophenotyping, but differences are also found in their biological properties such as proliferation, surface markers’ expression and differentiation potentials (7, 8). These discrepancies could contribute to cell fate determination and therapeutic efficacy after transplantation (9). For instance, PL-MSCs were superior in cell proliferation, survival, differentiation and immunomodulatory potentials compared to BM-MSCs (10). They possessed lineage differentiation capabilities beyond mesoderm such as myogenesis and neurogenesis when induced with respective media (11). Compared to ASCs, lesser risk in stimulating an allogeneic response after the administration of UC-MSCs as there was no increase in human leukocyte antigen-A, B, C (HLA-ABC) and HLA-DR expression after interferon-gamma stimulation (3, 12). Besides that, the paracrine factors like growth factors and cytokines secreted by MSCs from different compartment of perinatal tissues were distinct (13). For example, chorion plate-MSCs could be pro-angiogenesis as they secreted more hepatocyte growth factor (HGF) and vascular cell adhesion molecule 1 (VCAM-1) than amniotic membrane-MSCs with higher secretion of prostaglandin E2 (PGE2) and transforming growth factor beta 1 (TGF-β1).

Factors contributed to the differences of MSCs are the microenvironment, the localised function of each source and the ontogenetic age. However, it can also be caused by the process during isolation and culture condition (14, 15). Isolation techniques and culture conditions are also key determinants for the successful expansion of stem cells. In addition, disparities in morphology, immunophenotype, differentiation and the expression of pluripotency genes attributed by different isolation methods have also been widely reported. These have also contributed to variation in therapeutic outcomes observed through pre-clinical or clinical trials (9). Therefore, thorough considerations on the isolation method are pivotal before embarking on stem cell research to ensure reliable and unbiased interpretation of research findings. Here we review different types of MSC that can be derived from different compartments of perinatal tissues, their characteristics and the current approaches applied in isolating these MSCs.

### Human Placenta

**Figure 1:**
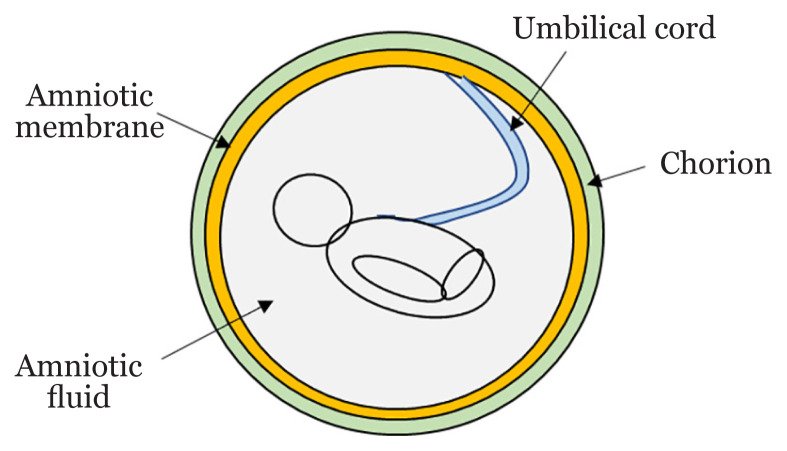
Schematic structure of the human PL

The human PL is rich with stem or progenitor cells and growth factors crucial for tissue repair and regeneration. The developing foetus is protected from external surroundings by a complex structure made of several membranes (Figure 1). PL is a foetomaternal organ where MSCs can be isolated from foetal (amnion, UC, chorion) or maternal (decidua) tissues (16). These two parts are connected by chorionic villi that holds chorionic sac and decidual basalis together. Foetus is separated from the endometrium because it is enclosed by the amniotic and chorionic membranes (16, 17). ESCs markers expressed in MSCs are c-KIT, OCT4, SOX2, SSEA3, SSEA4, TRA-1-60 and TRA-1-81. Besides mesoderm lineage differentiation, perinatal MSCs can also differentiate into endoderm and ectoderm lineage cells (18). Although MSCs derived from various compartments of PL are nondistinguishable via their morphology and immunophenotypes, tremendous studies have revealed that they are different in term of their gene expression profiles and differentiation capabilities. Table 1 summarised the characteristics and isolation methods of PL-MSCs mentioned in this manuscript.

### Amniotic Membrane

The foetal membrane consists of two distinct layers surrounding the foetus: amniotic membrane (AM) is the innermost layer of the PL. In contrast, chorionic membrane is at the outer surface of AM. Amnion is avascular tissue, primarily made of monolayer of amniotic epithelial cells (AECs) and amniotic mesoderm cells (AMCs) (19). AM has been applied in regenerative medicine and tissue engineering; mainly as an allograft for treatments such as burns, skin, corneal and dental transplantation. These are all attributed to their properties in wound healing, anti-inflammation and low immunogenicity. It can also act as scaffolds that partly promote cell proliferation and differentiation by secreting growth factors (20).

As both AECs and AMCs are originated from epiblast during gastrulation, they are believed to acquire stem cell-like properties. AECs can be isolated by trypsinization that removes these cuboid cells from the basal and mesenchymal layers after stripping the AM from the chorion. They have a cobblestone epithelial feature, which is different from the fibroblastic appearance of AM-MSCs (21). Both human amniontic epithelial stem cells (AESCs) and AM-MSCs expressed ESC surface markers such as SSEA-3, SSEA-4, SOX2, TRA-1-60, TRA-1-81, NANOG and OCT4 (19, 21). In addition to mesenchymal lineages, AECs could undergo tenogenesis both in vitro and in vivo (22).

Among the PL-MSCs, amnion membrane mesenchymal stem cells (AM-MSC) are the most well-studied source. AM-MSCs expressed high levels of typical MSCs markers but did not express hematopoietic markers (23). They can differentiate towards all three germ layers: ectodermal, mesodermal and endodermal lineage cells. Both pluripotency markers, octamer-binding transcription factor 4 (OCT4) and homeobox transcription factor NANOG (NANOG) are also expressed in AM-MSCs. Comparative analysis of several neonatal tissues in serum-free conditions showed that AM-MSCs exhibited the highest osteogenic potential compared to other MSCs (4).

### Chorion

Chorionic villi (CV) consist of four subtrophoblast layers that formed during the first trimester. Chorionic MSCs (Ch-MSCs) can be isolated from the CV and chorionic plate (CP). However, isolating these foetal MSCs is difficult due to maternal cell contamination or heterogeneous nature of the cell population harbouring this tissue as high incidence of maternal-origin MSC populations in Ch-MSC cultures has been reported (24–26). Thus, it is important to perform chromosome XY-fluorescence in situ hybridisation (XY-FISH) analysis to exclude the possibility of maternal contamination after cell isolation (25). Besides that, Abumaree et al. (27) demonstrated that CV-MSCs with mesoderm lineage differentiation expressed CD44, CD90, CD105, CD146, CD166 and HLA-ABC could be obtained using explant culture technique.

The contamination of maternal cells can be differentiated through their distinct features such as morphology, proliferation, adhesion and migration during in vitro expansion. Foetal CV-MSCs migrated faster and less adhesive than maternal cells (28). They were smaller in size and more proliferative and osteogenic than maternal cells (26). It has been reported that long-term culture of CV-MSCs maintained stable karyotype throughout 20 passages without affecting their mesodermal lineage differentiation. Persistent expression of pluripotency markers such as OCT4 and NANOG was observed up to 12th passage, but the expression of SOX2 was undetectable. MSC surface markers such as CD90, CD73, CD105, CD29, CD44, HLA ABC antigens were also consistently expressed after serial passaging (29). In contrast, a recent study showed that only pluripotency gene, SOX2 was expressed at both early and late passage of CV-MSCs. Surprisingly, the loss of telomere length in CV-MSCs was much lower than adipose and BM tissues despite the absence of human telomerase reverse transcriptase (hTERT) enzyme which is responsible for extending the end of chromosomes. This indicated that CV-MSCs might utilise other alternative mechanisms for lengthening their telomeres (30).

Torre and Flores (31) reviewed that MSCs derived from CP-MSC are foetal origin and found to express positive markers (CD73, CD90, CD105, CD44, CD166, CD106 and CD54) but negative for CD45, CD34, CD14, CD19 and HLA-DR. They showed superior proliferation, migration and immunomodulatory properties. Like other PL-MSCs, CP-MSCs could be differentiated towards various lineages such as adipogenesis, osteogenesis, chondrogenesis and hepatogenesis.

### Amniotic Fluid

Amniotic fluid (AF) is a protective layer that keeps the foetus safe by providing mechanical support and supplying nutrients during embryogenesis. The major component of AF is water, although its cellular composition always changes during pregnancy (32). AF represents a nourishing source of the stem cell population. However, collecting AF through amniocentesis is an invasive procedure that potentially causes foetal infection. Therefore, Caesarean section-derived AF has been used as an alternative source to obtain stem cells. Amniotic fluid cells are a population of heterogeneous cells that include cell types derived from epithelial surface, embryo itself or the inner amniotic surface. Evidence has shown that AF consists of differentiated cells, uncommitted or organ-committed progenitors and pluripotent cells which could be passaged for long-term up to 42 passages (33). Because of this heterogeneity, they are categorised based on morphology, growth and biochemical features. The amniotic fluid type (AF-type) represents 60% cells which are found to be the most abundant, less polar and have a higher growth rate. About 34% of them are epithelioid type (E-type) with large polygonal shape and moderate growth rate. The fibroblastic type (F-type, 4%) has a spindle shape and the highest growth rate (34). This has been ascribed to the different cellular origins of amniocytes with varying biological properties. Regardless of isolation methods, all of them are positive for CD44, CD29, CD90, CD105, OCT4, NANOG and SSEA4 (34). AF-MSCs were in closer similarity to adult stem cells compared to ESCs and induced pluripotency stem cells (iPSCs) (32). Extensive studies have demonstrated that cell number obtained through amniocentesis is mostly affected by volume, variation of donors and gestational stage. AF-MSCs isolated from the first trimester expressed pluripotency markers such as NANOG and SOX2 (35).

**Figure 2:**
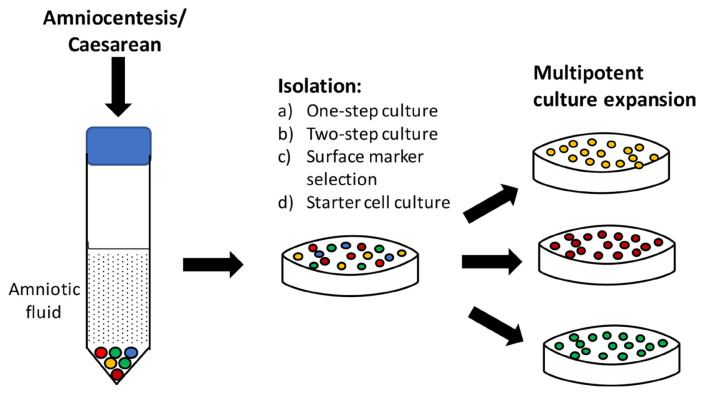
Isolation and cultivation of multipotent cells from amniotic fluid

As depicted in Figure 2, various isolation methods have been developed to ensure only potent MSCs are isolated (32). In the single-step procedure, amniocytes obtained is either cultured undisturbedly for 7 days or without changing medium for 20 days. While in the two-step protocol, non-adhesive amniocytes are transferred into a new plate for further cultivation after 5 days in culture medium. The selective isolation method is performed by sorting amniocytes expressing CD117 or CD133 positive surface markers. Then, the starter cell culture is collected by isolating fibroblastoid cell colonies for clonal expansion.

As obtaining AF-MSCs from the first and second trimester poses certain risks to the mother and foetus, the compatibility of cells from full-term pregnancy has been investigated as well by some researchers (11, 31). Moraghebi et al. (11) had demonstrated the feasibility of collecting a substantial volume of term amniotic fluid using a siphoning catheter-based system. They found spindle-shaped fibroblastic-like and round epithelioid-like cells exhibiting different proliferative activities in full-term AF-MSCs. Epithelioid-like cell population showed a slower proliferation rate than fibroblastic-like cells and could be cultured for long-term in vitro expansion. Reprograming these cells into hematopoietic and neural cell lineages is feasible (11). Thus, full-term AF could also be a promising source of mesenchymal stem cells.

### Umbilical Cord Blood

Umbilical cord blood (UCB) is considered one of the most suitable alternatives to obtaining MSCs for clinical applications (36). The isolation procedure is more straightforward than other tissues involving enzymatic digestion and explant culture. It has also been used in treating diseases such as haematological disorders and the UCB banking system for storage is also well established worldwide (37). Due to the low recovery of MSCs circulate in UCB, it is difficult to isolate and culture them compared to haematopoietic stem cells. Besides that, the yield is also inversely affected by gestation age (38). UCB-MSCs also underwent senescence earlier than other PL-MSCs. Nevertheless, they showed more prolonged survival and expansion potential than BM-MSCs or AT-MSCs (39). UCB-MSCs showed higher osteogenic potential than BM-MSCs, but they possessed comparable chondrogenic differentiation capacity. However, there are controversial findings about the differentiation ability of UCB-MSCs to adipocytes (39).

In general, there are two ways to collect cord blood samples: in utero (PL still in uterus) or ex utero (after PL delivery). The in utero approach is preferable because more cells can be collected through this sampling type and less contamination issue. However, relative low total nucleated cell (TNC) is obtained from these conventional approaches. Vanegas et al. (40) reported that the combined in/ex utero method not only increased the TNC count but also significantly reduced microbial contamination. In general, UCB can be collected by draining the blood from the cord or by needle aspiration of bare and engorged vessels. Increasing the dilution of CB will decrease the aggregation of mononuclear cells (MNCs) during density gradient centrifugation (41). The separation of MNCs is done by layering the diluted CB slowly on the top of Ficoll-paque solution followed by centrifugation at room temperature. The MNCs are carefully removed from the solution and washed several times using phosphate-buffered saline (41). The resulting cell pellets are suspended in culture medium and cultured until observing the outgrowth of fibroblastoid cells. UCB-MSCs strongly expressed CD90, CD105, CD44, CD13, and HLA-ABC, but they were negative for the haematopoietic markers such as CD31, CD34, CD45 and for HLA-DR (42).

One of the major setbacks contributing to the wide-ranging success rate between 10% and 90% in culturing UCB-MSCs is inconsistency in the isolation process (38, 42). Many researchers have collectively shown that sample volume, cellular content and processing duration after CB collection play an important role in determining the success rate (42, 43). However, Amati et al. (42) demonstrated that CB volume is not a determinant factor for the success of its MSC isolation. They found two populations with distinct proliferative, colony-forming and immunosuppressive capacity, which were termed as short- and long-living CB-MSCs.

### Umbilical Cord

**Figure 3:**
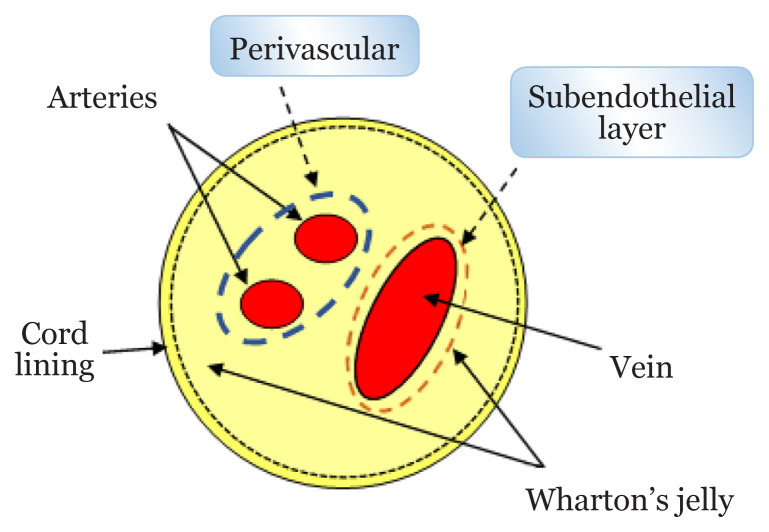
Structure of UC

The human UC is an increasingly popular cell source being developed for allogeneic cell-based therapy due to its superior immunopriviledged and immunomodulatory effects to other MSCs (44). UC is embryonic origin of tissue developed from the yolk sac and allantois which later becomes a conduit between mother and foetus (45). As shown in Figure 3, UC consists of two umbilical arteries and an umbilical vein surrounded and supported by jelly-like tissues known as Wharton’s jelly (WJ) and cord lining (46). MSCs can be obtained from UC, cord lining, WJ, perivascular region or subendothelial layer (47). UC-MSCs are generally referring to MSCs obtained from the whole areas of UC. WJ-MSCs are isolated from the WJ region after removing the cord lining, perivascular and subendothelial parts (45).

According to Semenova et al. (48), WJ-MSCs is the most promising among MSCs from other UC regions. WJ is an interior mass of mucoid connective tissue that lies between amniotic epithelium and umbilical vessels (46). The gelatinous matrix of WJ is made of glycosaminoglycan mainly hyaluronic acid and chondroitin sulfate, which forms strong ionic bonds with collagen fibres (46, 49). There are no capillaries and nervous system found in WJ (45, 46). WJ protects umbilical vessels by preventing them from compression, torsion and blending (45).

The primitive stem cells, such as haematopoietic cells and MSCs, are embedded in WJ. Some researchers used the whole piece of WJ for isolation. In contrast, others extracted cells from the cushioning matrix between the umbilical veins and two arteries located in the UC, but not the UCB (49). Studies showed that MSCs-derived from arterial, venous and WJ compartments showed similar phenotype with little differences in osteogenic potential. WJ-MSCs are found to have higher proliferation and better in vitro expansion capacity than the gold standard, BM-MSCs (44, 50). WJ-MSCs express positive stem cell marker including CD90, CD73, CD105 but do not express haematopoietic lineage markers, CD45 and CD34. They also express integrin markers CD29 and CD51 (49). The expression of pluripotency genes such as OCT4, SOX2, NANOG and REX-1 have been reported in WJ-MSCs, but their expression level is relatively lower than ESCs (48, 51). Vimentin, a type of intermediate filament protein, is also highly expressed in WJ-MSCs (52). Besides having trilineage differentiation capabilities like BM-MSCs, they have been successfully differentiated into hepatocyte-like cells (53), neuron-like cells (54) and cardiac-like cells (55). Numerous preclinical and human clinical trials have been conducted using WJ-MSCs for regenerative and reconstructive medicine including neurological, liver, cardiac, haematological, immunological, endocrine and musculoskeletal disorders (56).

Besides that, it is worth mentioning that the lack of terminological standardisation has led to the difficulty in classifying MSCs based on the anatomical regions of UC. We noticed that sometimes MSCs derived from WJ were generally termed UC-MSCs especially when there is no description of where they are isolated. This misconception can mislead and jeopardise the interpretation of scientific data, especially when comparing the efficacy of MSCs for clinical use.

### Isolation of Mesenchymal Stromal/Stem Cells

**Figure 4:**
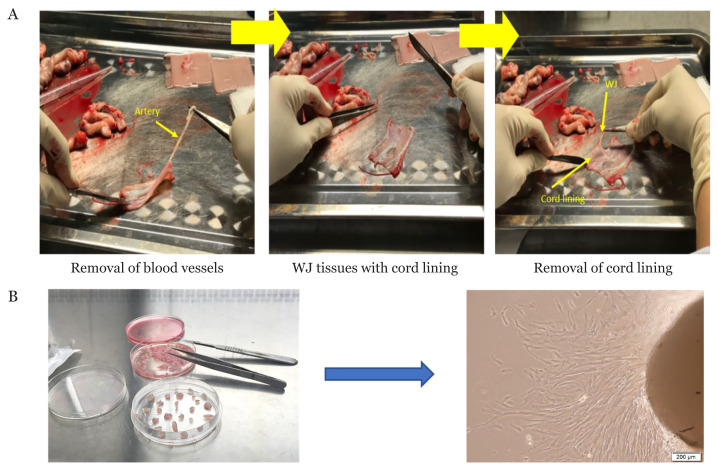
Isolation of MSCs. (A) Processing of umbilical cord and (B) Explant culture technique

In general, the isolation methods for perinatal tissues are quite similar, except for UCB. Enzymatic treatment and explant culture are commonly used for the isolation of MSCs, although perfusion and flow cytometry can also be used (57, 58). In this review, we focused on two common methods as cell viability is higher compared to others. Freshly collected UC is processed immediately within 48 h (59) but for tissue bank, explant method is preferable whereby UC is stored in liquid nitrogen before processing (57). Firstly, both arteries and vein are removed from UC before cutting them into small pieces, but some use whole UC or different parts of UC (60). After carefully peeling the cord lining from WJ (Figure 4A), the tissue is cut into small pieces (57). For the explant culture method (Figure 4B), tissues are placed on a culture dish and cultured in media supplemented with antibiotics and growth factors containing serum (60–63). Attachment-promoting substrate and stainless-steel mesh can be used to enhance attachment and avoid floating of explant tissue (57, 64). For enzymatic method, tissues are subjected to one or combination of enzyme(s) such as trypsin, hyaluronidase or collagenases (I, II, IV) before culturing (59, 62, 65). After digestion, cell pellet is collected by centrifugation and then resuspended with culture media such as Dulbecco’s modified Eagle’s medium (DMEM) or DMEM/F12 and ready to be cultured at 37 °C in 5% CO_2_ incubator (66, 67). Cells normally reach 80% confluency after 7 days–14 days depending on cell type and the isolation method while explant method needs longer time.

Some studies reported that the enzymatic method affects cell proliferation and viability because of the long incubation period and relies on the type of enzyme and parameters that influence the efficiency of catalytic activity such as time, enzyme-substrates ratio and temperature (61, 62). By skipping the above incubation step, explant culture is faster than enzymatic method and can avert potentially cellular damage caused by proteolytic enzymes (60, 62). Besides that, the extracellular matrix or cellular components that are still attached to the explant will continue to provide substances that benefit the growth of migrating cells (60, 68). Among the disadvantages of explant culture are migrated MSCs from tissue needs longer time to reach confluence, inability to calculate the number of isolated cells, inconsistency of MSC migrating out from explant tissue and low recovery rate when explant is not attached to the surface (57, 64). There are studies reported explant culture showed significant variation in the expression of surface molecules compared to the enzymatic method (69). On the contrary, comparable findings have been reported on proliferation, immunophenotype and differentiation properties for these two methods (57). Salehinejad et al. (61) optimised the incubation period of three selected enzymes before mixing them to find the best combination for cell isolation. Besides that, mixed mechanical and enzymatic methods have shown to increase cell yield. For example, mechanical dissociation was performed by adding enzymes into the tissue and then incubated for 3 h–3.5 h at 37 °C with 12 rpm rotation (70). Mixed enzymatic-explant method has been shown to produce more homogenous MSC population with less damage. This can be carried out by incorporating mild enzymatic steps before the explant culture (24, 71). To increase the yield of MSCs from extraembryonic tissues, various optimisations have also been incorporated during isolation including types of enzymes, and their effectiveness has been reviewed by Salehinejad et al. (62).

Recently, Yi et al. (58) demonstrated that the cellular components of tissues could affect the choice of isolation methods. They compared MSCs isolated from PL and UC by processing these tissues using a homogenizer before proceeding to explant, enzymatic or perfusion methods. However, the perfusion technique yielded low cell number and high heterogeneity besides being complicated and time-consuming. The enzymatic method was the most suitable for AM because its texture is smooth and thin thus having difficulty to attach onto plastic surface. Same as CV, the efficiency was low with explant method due to the complex structure of this tissue. In contrast, explant technique was recommended for UC.

However, the drawbacks of selection based on cell adhesive ability on plastic followed by passaging using serum supplemented media are the cellular heterogeneity at early passage and loss of MSC markers after in vitro expansion. The variable composition of not well-defined serum can also cause differences in MSC growth and quality. Moreover, this animal derived component may pose contamination risk and provoke immune response during clinical use. To ameliorate these adverse consequences, serum-free or xeno-free media and the use of biomimetic surface have been adopted in MSC isolation and expansion (38, 58, 72). Nonetheless, in vitro expansion using serum-free media is cell type-specific and not all available serum-free media in the markets are compatible for MSCs from different sources. Not only that, some MSCs may require additional coating agents for supporting cell adhesion (38, 73, 74). Thus, the suitability of serum-free media for a specific MSC expansion needs substantial analysis on MSC characteristics to ensure its therapeutic function is preserved before large-scale production.

### Limitations of Mesenchymal Stromal/Stem Cells for Clinical Applications

Despite the outstanding therapeutic outcomes from preclinical investigations, most of the clinical trials could not be reached at a later stage. The low success rate is due to heterogeneity of MSCs attributed partly by the isolation and expansion methods which are unable to avoid mixed cell population (75, 76). Besides that, MSC characterisation based on the minimal criteria set by ISCT is insufficient to distinguish different types of stem-like or progenitor cells. There is no single unique surface marker for identifying each type of MSC from different origins has also jeopardized the procurement of ‘pure’ MSC. In addition, lack of standardisation in the isolation and culturing protocols among laboratories is also one of many reasons. This is further complicated by the fact that the therapeutic potential of MSC is also cell-type dependent.

Both PL and UC are promising sources of MSCs, consensus on understanding their complex tissue structure is important as diverse cell populations can be derived from the same or different regions. It is suggested that isolation techniques based on anatomical structure described by Silini et al. (16) should be followed in order to acquire genuine stem cell types for cell therapy. Besides that, comparative characterisation of MSCs from various PL compartments also showed that they possess different gene and protein expression profiles regardless of serum or serum-free condition. These discrepancies could originate from heterogenous cell populations and varied isolation or expansion methods which have implications on MSC functionality in clinical settings (77).

Thus, concerted efforts towards standardisation on the isolation and processing of MSC should be emphasised internationally. This will ease the comparison of MSC research across different laboratories and a stepping-stone for ensuring the high quality of MSC production that meets the regulatory standards for clinical use (76, 78).

### Future Perspectives

The considerable interest and applications of MSCs derived from perinatal tissues will undeniably continue growing in various aspects not limited to regenerative medicine and tissue engineering. They have also proven promising for infectious diseases such as COVID-19 recently due to their excellent immunomodulatory potential, although more research is required to further understand the mode of action. However, some challenges still need to be resolved especially safety concerns and consistency supply to guarantee their clinical efficacies. Standardising isolation techniques and in vitro expansion for each cell type from different sources is crucial also because this is one of the key factors influencing self-renewal and differentiation properties.

MSCs derived from different compartments of perinatal tissues are also found to have distinct biological properties which impact their differentiation potentials in clinical settings. Besides a comprehensive understanding of their intrinsic properties through multi-omics approaches, integrated interdisciplinary research such as biomaterials and nanotechnology will help in modulating and directing cell fate towards desired specifications for therapeutic purposes. This ultimately bridges the gap between the fundamental knowledge and translational application of these MSCs.

## Figures and Tables

**Table 1:** The characteristics and isolation methods of MSCs from different sources

Source		Characteristic		Isolation method

		Morphology	Surface markers	
Amnion membrane	Amniotic epithelial stem cells	Cobblestone epithelial	SSEA-3, SSEA-4, SOX2, TRA-1-60, TRA-1-81, NANOG, OCT4	Enzymatic digestion or explant culture

	Amniotic membrane MSCs	Fibroblastic		
Chorion	chorionic villi MSCs	Fibroblastic	CD44, CD90, CD73, CD29, CD105, CD146, CD166, HLA-ABC	Enzymatic digestion or explant culture

	chorionic plate MSCs	Fibroblastic	CD73, CD90, CD105, CD44, CD166, CD106, CD54	
Amniotic fluid	AF-type	Spindle shape	CD44, CD29, CD90, CD105, OCT4, NANOG, SSEA4	Amniocentesis or Caesarean section followed by either single-step cultivation, two step cultivation or c-kit (CD117) selection

	E-type	Large polygonal shape		

	F-type	Spindle shape		
Umbilical cord	Cord blood MSCs	Fibroblastic	CD90, CD105, CD44, CD13, HLA-ABC	Blood is collected either in utero or ex utero then MNCs are separated in Ficoll-paque solution using density gradient centrifugation

	Wharton’s jelly MSCs	Fibroblastic	CD90, CD73, CD105, OCT4, SOX2, NANOG, REX-1	Enzymatic digestion or explant culture
